# Association of white matter hyperintensity with systemic inflammation markers and cognitive assessments: a cross-sectional study via SHAP analysis

**DOI:** 10.3389/fnagi.2025.1667025

**Published:** 2025-11-28

**Authors:** Dewang Gao, Jiayu Lv, Xinhui Li, Wen Yong, Wenlong Yu, Lu Wang, Shangjia Ma, Hua Li, Shuaiqiang Zhang, Zi Guo, Hao Yan, Zhipeng Ju, Yiming Liu, Xia Guo, Lie Wu

**Affiliations:** 1 Department of Neurology, The First Affiliated Hospital of Baotou Medical College, Baotou, China; 2 BaogangHospital of Inner Mongolia, Baotou, China

**Keywords:** white matter hyperintensity, Montreal Cognitive Assessment, systemic inflammation response index, model comparison and internal validation, SHAP analysis

## Abstract

**Background:**

White matter hyperintensity (WMH), a common neuroimaging feature in the older adults, has not been systematically elucidated regarding its association with cognitive function and systemic inflammation.

**Aim:**

To develop and validate a clinical model for higher WMH burden integrating MoCA and CBC-derived inflammatory markers, and to quantify their independent and joint associations with WMH severity.

**Methods:**

This study retrospectively collected data from patients with WMH at the First Affiliated Hospital of Baotou Medical College (December 2023–December 2024). We used univariate and multivariate logistic regression analyses to identify WMH-related variables. Then, we constructed an artificial neural network model and performed 10-fold cross-validation for internal validation and model performance comparison. The Shapley Additive Explanations (SHAP) method was employed to evaluate both models.

**Results:**

Correlation analysis revealed a significant association between the systemic inflammation response index (SIRI) and WMH burden (*P*< 0.01). Multivariate logistic regression analysis identified age, hypertension, high-density lipoprotein (HDL), previous cerebrovascular disease, the systemic inflammation response index (SIRI), and the Montreal Cognitive Assessment (MoCA) score as independent predictors of WMH burden. Ten-fold cross-validation showed that the set neural network model performed as well as the logistic regression model (AUC = 0.81). SHAP-based visual analysis identified age, MoCA score, and hypertension as key driving factors.

**Conclusion:**

Age, hypertension, previous cerebrovascular disease, HDL, SIRI, and MoCA score are independent risk factors for moderate to severe WMH occurred. The model integrating MoCA and inflammatory markers accurately predicts moderate to Severe WMH. This study offers a multidimensional assessment framework for WMH risk stratification and early intervention.

## 1 Introduction

White matter hyperintensity (WMH) is a significant neuroimaging marker on brain magnetic resonance imaging (MRI), appearing as characteristic hyperintensities on T2-weighted and fluid-attenuated inversion recovery (FLAIR) sequences. Its underlying pathology includes demyelination, axonal degeneration, and gliosis (Karvelas and Elahi, 2023). Epidemiological studies demonstrate a marked increase in WMH prevalence with age: ranging from 11 to 21% in healthy individuals aged 64 years (Debette and Markus, 2010) to 94% in those aged 82 years. Among individuals with vascular risk factors, WMH prevalence is 68–87% in those aged 60–70 years, rising to 95% for periventricular WMH (PVWMH) in octogenarians and nonagenarians (80–90 years) (da Silva et al., 2022; Debette and Markus, 2010). Individuals with WMH have a 2.6–4.4 times higher risk of stroke, a 1.3–2.8 times greater risk of dementia, and nearly twice the risk of mortality compared to those without WMH (Debette and Markus, 2010). Studies have confirmed that WMH volume is positively correlated with cognitive impairment (da Silva et al., 2022). Furthermore, rapid WMH progression has been established as a significant predictor for the transition from preclinical Alzheimer’s disease to clinical dementia (Egle et al., 2022; Gu et al., 2022; Saleh et al., 2021; Tully et al., 2020). Longitudinal studies suggest spatial heterogeneity in WMH progression, with annual increases of 0.2% in subcortical and 0.4% in periventricular regions (Salvadó et al., 2019).

WMH represents a characteristic neuroimaging manifestation of cerebral small vessel disease (CSVD). Its etiology is multifactorial, encompassing demyelination, axonal injury, and vascular pathology secondary to chronic hypoperfusion. These pathological alterations disrupt neural connectivity and ultimately lead to cognitive decline. Age and hypertension are established independent risk factors (Salvadó et al., 2019), while non-traditional factors such as metabolic dysregulation and sleep-disordered breathing also contribute to the pathological process (Salvadó et al., 2019). The pathogenesis of vascular-related WMH likely involves a synergistic interaction among cerebral hypoperfusion, blood–brain barrier (BBB) disruption, and oxidative stress (Karvelas and Elahi, 2023). Emerging evidence highlights the pivotal role of neuroinflammation in WMH pathogenesis: BBB disruption facilitates infiltration of peripheral immune cells and release of pro-inflammatory cytokines, initiating a vicious cycle of inflammation-endothelial damage-ischemia (Jiang et al., 2022). Animal studies confirm that spontaneously hypertensive rats (SHRs) recapitulate the neuroinflammatory features and white matter damage observed in patients with CSVD (Kaiser et al., 2014). Genetic studies support the involvement of inflammatory pathways (Jickling et al., 2022). Meanwhile, readily accessible complete blood count-derived inflammatory indices, such as the systemic immune-inflammation index [SII (neutrophils × platelets/lymphocytes)] and neutrophil-to-lymphocyte ratio [NLR (neutrophils/lymphocytes)], its accessibility provides a new idea for WMH evaluation (Del Brutto et al., 2023; Jiang et al., 2022; Liang et al., 2023; Nam et al., 2022; Wang et al., 2022).

However, current clinical practice faces dual challenges: the high cost of multi-sequence MRI restricts its widespread application in primary care settings, and the precise mechanisms linking inflammatory markers to WMH remain incompletely elucidated. This study aims to systematically explore the relationship between systemic inflammatory markers (including SII, NLR, etc.) and WMH burden, construct a predictive model integrating clinical data and inflammatory indices, and thereby provide an evidence-based foundation for early WMH screening and intervention. The findings provide an important basis for optimizing diagnostic-therapeutic pathways and promoting the clinical translation of community-based WMH prevention and management strategies.

## 2 Materials and methods

### 2.1 Research design and participants

This retrospective study enrolled 327 patients who visited the Department of Neurology at the First Affiliated Hospital of Baotou Medical College between December 2023 and December 2024. Inclusion criteria were: (1) Presence of WMH on brain MRI; (2) Age ≥ 40 years; (3) Availability of complete laboratory and imaging data. Exclusion criteria comprised: (1) Concurrent hematological disorders, acute infectious diseases, or other significant systemic illnesses; (2) A history of acute cerebral infarction (within 14 days), acute cerebral hemorrhage, aneurysms, or arteriovenous malformations; (3) Non-vascular white matter pathologies (e.g., multiple sclerosis, encephalitis, toxic leukoencephalopathy, hydrocephalus, traumatic brain injury); (4) Epilepsy, malignant tumors, or severe cardiac, pulmonary, hepatic, or renal dysfunction; (5) Inability to cooperate with cognitive function assessment during the study period. The study protocol was approved by the Ethics Committee of the First Affiliated Hospital of Baotou Medical College (Approval No. 2024-K044-01). All procedures were conducted in strict accordance with ethical guidelines, and we applied for exemption from written informed consent.

### 2.2 Data collection and systemic inflammatory marker assessment

Comprehensive demographic data, including age, sex, and lifestyle factors, were collected for each patient. Laboratory tests at admission included complete blood count, fasting plasma glucose (FPG), low-density lipoprotein cholesterol (LDL-C), and other relevant parameters. Cognitive function was assessed using the Montreal Cognitive Assessment (MoCA) (Salvadori et al., 2021). Several systemic inflammatory markers were calculated, including: neutrophil-to-lymphocyte ratio (NLR = neutrophil count/lymphocyte count), derived neutrophil-to-lymphocyte ratio [dNLR = neutrophil count/(white blood cell count − neutrophil count)], lymphocyte-to-monocyte ratio (LMR = lymphocyte count/monocyte count), platelet-to-lymphocyte ratio (PLR = platelet count/lymphocyte count), systemic immune-inflammation index (SII = neutrophil count × latelet count/lymphocyte count), lymphocyte-to-white blood cell ratio (LWR = lymphocyte count/white blood cell count), neutrophil-to-white blood cell ratio (NWR = neutrophil count/white blood cell count), platelet-to-neutrophil ratio (PNR = platelet count/neutrophil count), systemic inflammation response index [SIRI = (neutrophil count × monocyte count)/lymphocyte count], and eosinophil-to-lymphocyte ratio (ELR = eosinophil count/lymphocyte count). All data were collected by trained neurologists at the First Affiliated Hospital of Baotou Medical College, with strict confidentiality measures implemented to protect participant information throughout and after data collection.

### 2.3 Cognitive assessment

MoCA evaluated eight cognitive domains: visuospatial/executive function, naming, memory, attention, language, abstraction, delayed recall, and orientation. Total score: 30 points; ≥26 indicated normal cognition. One point was added for individuals with ≤12 years of education. Assessments were completed within 3 days of enrollment by trained neuropsychologists.

### 2.4 MRI acquisition and evaluation

Brain MRI was conducted using a 3.0T scanner with sequences including T1-weighted imaging (T1WI), T2-weighted imaging (T2WI), diffusion-weighted imaging (DWI), fluid-attenuated inversion recovery (FLAIR), and susceptibility-weighted imaging (SWI). Two experienced neuroradiologists, blinded to clinical data, independently rated WMH burden using the Fazekas scale. Inter-rater agreement for categorical scores was evaluated using weighted kappa (κ), while the reliability of continuous measurements was assessed with intraclass correlation coefficients (ICC). All MRI scans were independently evaluated according to the most recent expert consensus (Duering et al., 2023). Any discrepancies were resolved through consensus discussion or adjudication by a third expert. WMH lesions were defined as punctate or patchy hyperintensities on T2WI/FLAIR sequences, appearing isointense or hypointense on T1WI without cavitation, typically exhibiting bilateral symmetric distribution. WMH severity was graded according to the Fazekas scale:

- Periventricular WMH (PVWMH): 0 (none), 1 (caps or pencil-thin lining), 2 (smooth halo), 3 (irregular extension into deep white matter)

- Deep WMH (DWMH): 0 (none), 1 (punctate foci), 2 (beginning confluence), 3 (large confluent areas)

**FIGURE 1:**
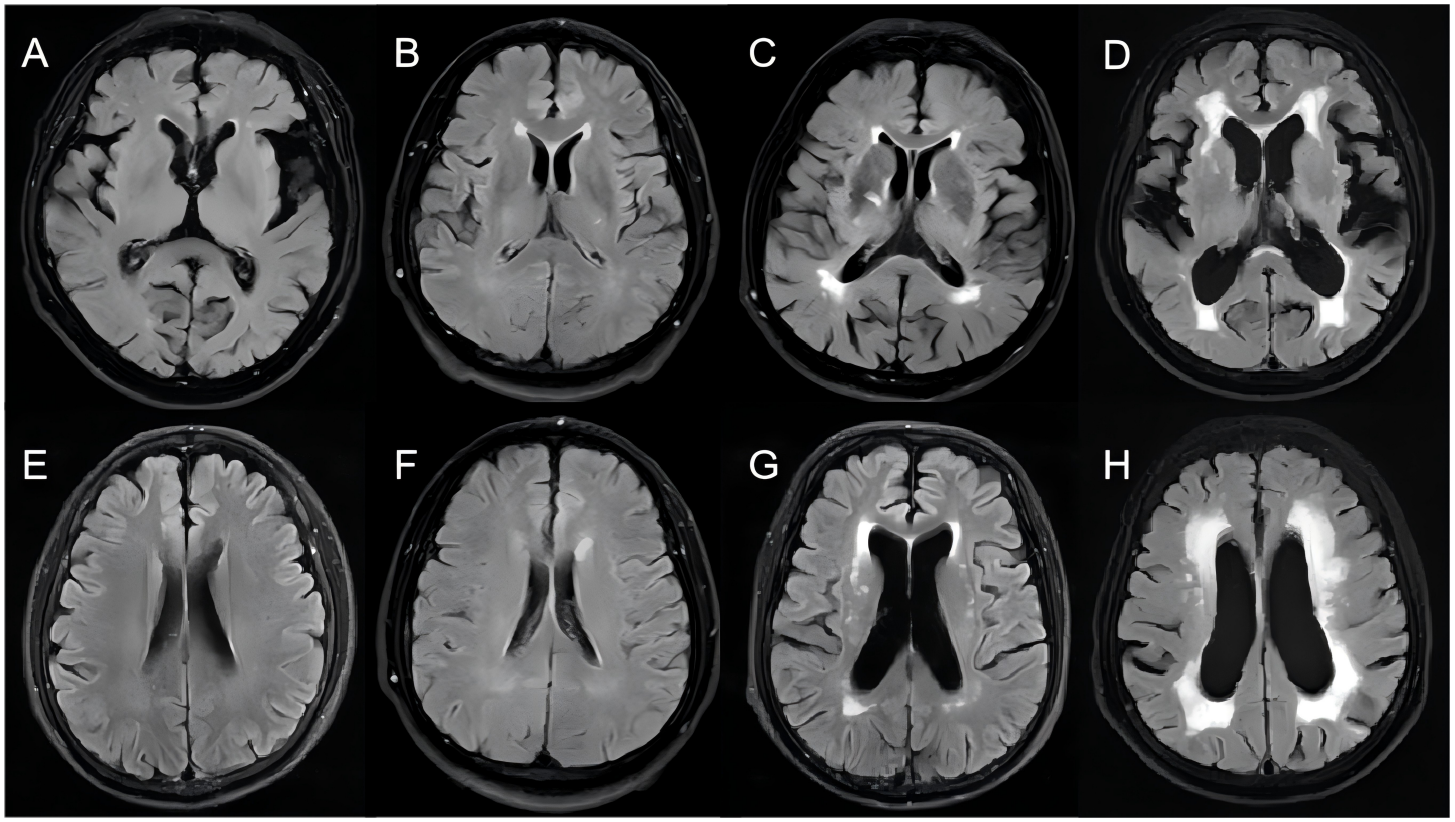
T2 FLAIR imaging reports of head MRI. Periventricular WMH (PVWMH) fazekas scores: **(A)** 0 points, **(B)** 1 point, **(C)** 2 points, **(D)** 3 points; Deep WMH (DWMH) Fazekas scores: **(E)** 0 points, **(F)** 1 point, **(G)** 2 points, **(H)** 3 points.

Total Fazekas scores (0–6) were used to classify patients into mild (0–2) and moderate-to-severe (3–6) WMH groups (Figure 1).

### 2.5 Model development

**FIGURE 2:**
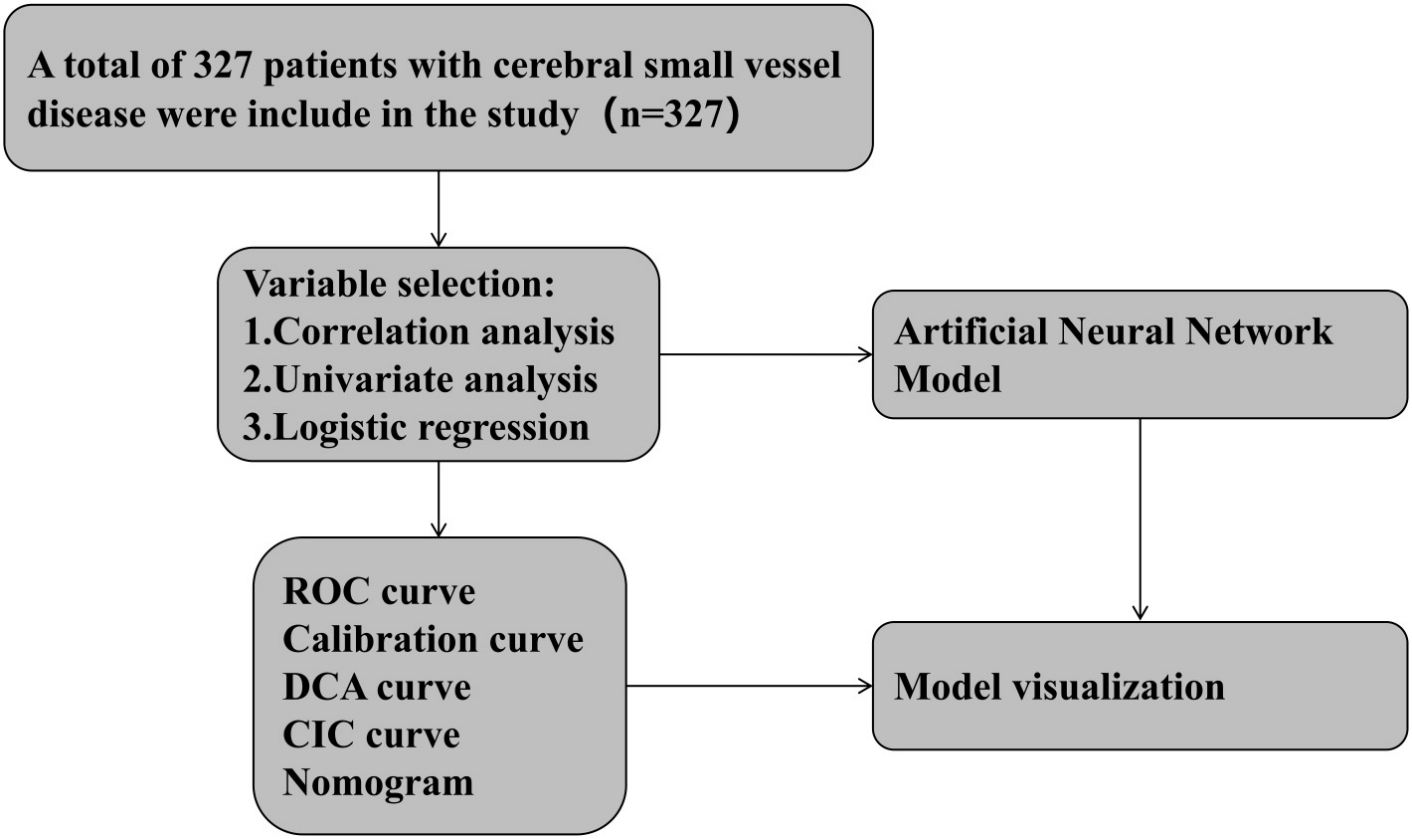
The flow chart of this study. ROC, receiver operator characteristic; DCA, decision curve analysis; CIC, clinical impact curve.

Univariate and multivariate logistic regression analyses were performed for variable selection. Variance inflation factor (VIF) analysis was applied to control multicollinearity, retaining variables with VIF < 5. The predictive model was evaluated based on the area under the receiver operating characteristic curve (AUC), calibration curves, decision curve analysis (DCA), and clinical impact curves (CIC) to assess its predictive performance and clinical utility. We further developed an artificial neural network model based on independent predictors from the logistic regression model, and conducted internal validation and model performance comparison. Model interpretability was examined using SHapley Additive exPlanations (SHAP), including feature importance rankings, beeswarm plots, and individual force plots. The complete experimental procedure is shown in Figure 2.

### 2.6 Statistical analysis

Data analysis and visualization were conducted using SPSS version 26.0 and R version 4.2.2. Continuous variables with normal distribution were expressed as mean ± standard deviation (SD) and compared using independent *t*-tests. Non-normally distributed and ordinal variables were presented as median (interquartile range, IQR) and compared using the Mann–Whitney U test. Spearman’s rank correlation was used to assess the relationships among inflammatory markers, imaging features, and cognitive scores. Backward stepwise binary logistic regression was used to identify independent risk factors. Receiver operating characteristic (ROC) analysis was conducted to evaluate predictive performance, and a nomogram was constructed. Based on the independent factors identified from the complete dataset, a single-hidden-layer feedforward neural network was constructed with the following specifications: 3 hidden nodes, weight decay regularization (decay factor = 0.1), and a maximum of 1,000 training iterations. The discriminative performance of neural networks and logistic regression models was evaluated using the ROC curve, which was plotted based on 10-fold cross-validation results. The AUC and its 95% confidence interval were employed to quantify model performance. The DeLong test was applied to compare ROC curve differences between neural networks and logistic regression models, with a significance level set at α = 0.05. Additionally, SHAP was utilized to quantify the contribution of each feature to the model predictions, with statistical significance defined as *P* < 0.05. R packages including pROC, CBCgrps, rms, and rmda were employed for data processing and visualization.

## 3 Results

### 3.1 Clinical characteristics of WMH patients

A total of 327 patients with WMH were enrolled, including 152 with mild WMH and 175 with moderate-to-severe. No significant differences were observed between WMH severity groups in terms of sex, smoking status, alcohol consumption, or triglyceride levels (*P* > 0.05). However, significant differences were observed in age, and the prevalence of diabetes, hypertension, and cognitive impairment between the two groups (*P*< 0.05). Compared to the mild WMH group, the moderate-to-severe group showed significantly lower levels of low-density lipoprotein cholesterol (LDL-C, *p* = 0.004), high-density lipoprotein cholesterol (HDL-C, *p* = 0.005), total cholesterol (TC, *p* < 0.001), lymphocyte-to-monocyte ratio (LMR, *p* < 0.001), and platelet-to-neutrophil ratio (PNR, *p* = 0.019), along with significantly higher levels of fasting plasma glucose (FPG, *p* = 0.005), uric acid (UA, *p* = 0.009), neutrophil-to-lymphocyte ratio (NLR, *p* = 0.029), and systemic inflammation response index (SIRI, *p* = 0.002) (Table 1).

**TABLE 1:** Baseline characteristic of the study subject.

Variables	Total (*n* = 327)	Mild (*n* = 152)	Moderate-severe (*n* = 175)	Statistic	*P*
**Demographic features**					
Male, n(%)	162 (49.54)	67 (44.08)	95 (54.29)	χ^2^ = 3.39	0.066
Age, years	66.18 ± 9.17	63.32 ± 8.36	68.67 ± 9.14	*t* = –5.49	<0.001
[40,56]	45 (13.76)	29 (19.08)	16 (9.14)	χ^2^ = 16.05	<0.001
[57,73]	203 (62.08)	100 (65.79)	103 (58.86)		
[74,90]	79 (24.16)	23 (15.13)	56 (32.00)		
Smoking, n(%)	76 (23.24)	28 (18.42)	48 (27.43)	χ^2^ = 3.70	0.054
Alcohol history, n(%)	43 (13.15)	20 (13.16)	23 (13.14)	χ^2^ = 0.00	0.997
Previous cerebrovascular disease, n(%)	93 (28.44)	19 (12.50)	74 (42.29)	χ^2^ = 35.46	<0.001
**Metabolic related indicators**					
Hypertension, n(%)	201 (61.47)	75 (49.34)	126 (72.00)	χ^2^ = 17.63	<0.001
BMI	24.98 ± 3.51	24.73 ± 3.56	25.19 ± 3.46	*t* = –1.17	0.242
Diabetes, n(%)	81 (24.77)	26 (17.11)	55 (31.43)	χ^2^ = 8.96	0.003
LDL, mmol/L	2.56 (2.08, 3.19)	2.76 (2.21, 3.41)	2.45 (1.98, 3.05)	*Z* = –2.85	0.004
HDL, mmol/L	1.11 (0.94, 1.31)	1.14 (0.98, 1.34)	1.08 (0.91, 1.24)	*Z* = –2.83	0.005
FPG, mmol/L	5.50 (4.90, 6.40)	5.30 (4.80, 6.03)	5.60 (5.00, 6.85)	*Z* = –2.80	0.005
TC, mmol/L	4.17 (3.52, 4.97)	4.46 (3.76, 5.13)	3.97 (3.33, 4.79)	*Z* = –3.37	<0.001
TG, mmol/L	1.44 (0.99, 2.02)	1.38 (0.99, 2.08)	1.44 (1.00, 1.98)	*Z* = –0.14	0.886
UA, μmol/L	323.00 (267.00, 387.50)	313.50 (249.75, 367.75)	340.00 (277.00, 399.50)	*Z* = –2.61	0.009
**Systemic inflammatory markers**					
Neutrophils, × 10^∧^9/L	4.26 (3.23, 5.37)	4.18 (3.05, 5.23)	4.28 (3.44, 5.47)	*Z* = –1.00	0.316
Lymphocytes, × 10^∧^9/L	1.54 (1.20, 2.00)	1.67 (1.23, 2.07)	1.50 (1.18, 1.89)	*Z* = –1.78	0.076
Platelets, × 10^∧^9/L	212.00 (171.50, 251.00)	216.50 (178.00, 258.25)	206.00 (167.00, 243.00)	*Z* = –2.24	0.025
WBC, ×10^∧^9/L	6.55 (5.50, 7.76)	6.49 (5.42, 7.83)	6.59 (5.61, 7.72)	*Z* = –0.56	0.575
HGB, g/L	143.00 (134.00, 155.00)	143.00 (135.75, 152.25)	143.00 (132.00, 157.50)	*Z* = –0.20	0.844
Monocyte, ×10^∧^9/L	0.43 (0.34, 0.54)	0.39 (0.32, 0.52)	0.47 (0.35, 0.58)	*Z* = –3.20	0.001
Eosinophils, ×10^∧^9/L	0.07 (0.03, 0.14)	0.07 (0.04, 0.14)	0.07 (0.03, 0.13)	*Z* = –0.13	0.899
Basophils, × 10^∧^9/L	0.03 (0.02, 0.04)	0.03 (0.01, 0.04)	0.03 (0.02, 0.04)	*Z* = –1.04	0.298
NLR	2.62 (1.89, 3.73)	2.42 (1.73, 3.49)	2.80 (2.02, 3.89)	*Z* = –2.19	0.029
dNLR	0.65 (0.59, 0.72)	0.64 (0.57, 0.72)	0.67 (0.60, 0.73)	*Z* = –1.62	0.106
PLR	133.06 (104.68, 173.14)	133.73 (104.71, 175.75)	132.16 (104.96, 172.71)	*Z* = –0.26	0.795
LMR	3.64 (2.68, 4.88)	4.12 (3.00, 5.06)	3.30 (2.48, 4.67)	*Z* = –3.94	<0.001
LWR	0.25 (0.19, 0.31)	0.26 (0.20, 0.33)	0.24 (0.19, 0.29)	*Z* = –2.40	0.017
NWR	0.65 (0.59, 0.72)	0.64 (0.57, 0.72)	0.67 (0.60, 0.73)	*Z* = –1.62	0.106
PNR	50.87 (37.39, 64.47)	53.75 (40.51, 68.44)	48.23 (36.89, 60.57)	*Z* = –2.34	0.019
ELR	0.05 (0.02, 0.08)	0.04 (0.02, 0.08)	0.05 (0.02, 0.08)	*Z* = –0.73	0.466
SII	533.43 (377.90, 815.03)	515.83 (363.12, 808.27)	542.15 (393.02, 815.03)	*Z* = –0.74	0.457
SIRI	1.15 (0.73, 1.76)	0.99 (0.67, 1.50)	1.27 (0.82, 1.82)	*Z* = –3.08	0.002
**Cognition and emotion**					
MOCA	22 (15, 27)	25 (21, 27)	17 (12, 23)	*Z* = –7.80	<0.001

Values for continuous variables are expressed as mean ± standard deviation or interquartile range; values for categorical data are given as numbers (percent). Mild: WMH total score ≤2 points. Moderate-Severe: WMH total score >2 points. NLR, Neutrophil Count/Lymphocyte Count; dNLR, Neutrophil Count/(White Blood Cell Count - Neutrophil Count); LMR, Lymphocyte Count/Monocyte Count; PLR, Platelet Count/Lymphocyte Count; SII, Neutrophil Count × Platelet Count/Lymphocyte Count; LWR, Lymphocyte Count/White Blood Cell Count; NWR, Neutrophil Count/White Blood Cell Count; PNR, Platelet Count/Neutrophil Count; SIRI, (Neutrophil Count × Monocyte Count)/Lymphocyte Count; ELR, Eosinophil Count/Lymphocyte Count; *p*-values are compared between Mild and Moderate-Severe groups. T, *t*-test; Z, Mann-Whitney test; χ^2^, Chi-square test.

### 3.2 Correlation analysis of WMH

**FIGURE 3:**
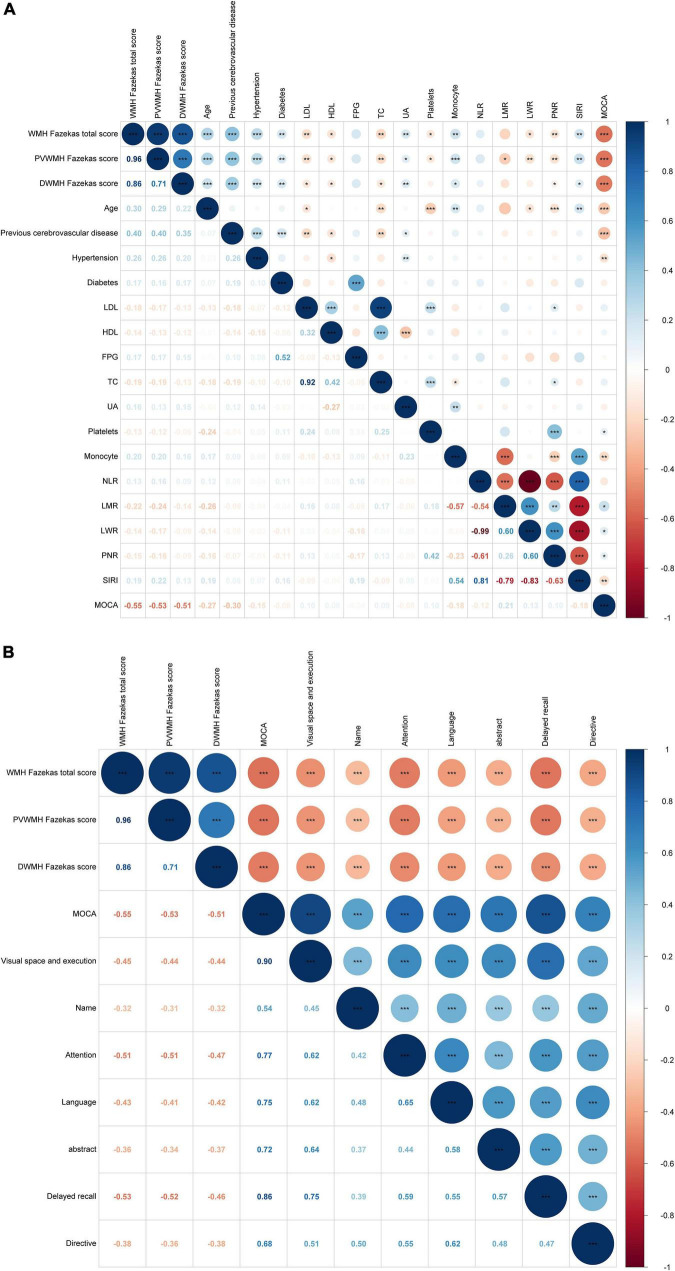
**(A,B)** Spearman correlation heat map of WMH variables. The upper triangle of the figure shows the significant relationship between each variable, and the lower triangle shows the correlation coefficient. The size of the circle represents the correlation coefficient, and the depth of color represents the correlation coefficient (*r*-value), * <0.05; ** <0.01; *** <0.001.

Two neurologists assessed the WMH burden. Inter-rater agreement was excellent (weighted κ = 0.91, 95% CI 0.89–0.94, *p* < 0.001; ICC = 0.98, 95% CI 0.98–0.99, *p* < 0.001), ensuring consistent WMH classification. Correlation analysis of 17 differentially expressed variables revealed that WMH burden showed a significant positive correlation with Siri (*r* = 0.19, *P*<0.01) and a significant negative correlation with MoCA scores (*r* = –0.55, *P*<0.001). However, no significant correlation was found between fasting plasma glucose (FPG), NLR, and LMR and WMH (*p*> 0.05) (Figure 3A). Figure 3B illustrates the correlation between WMH scores and various subgroups in the MOCA.

### 3.3 Feature selection

**FIGURE 4:**
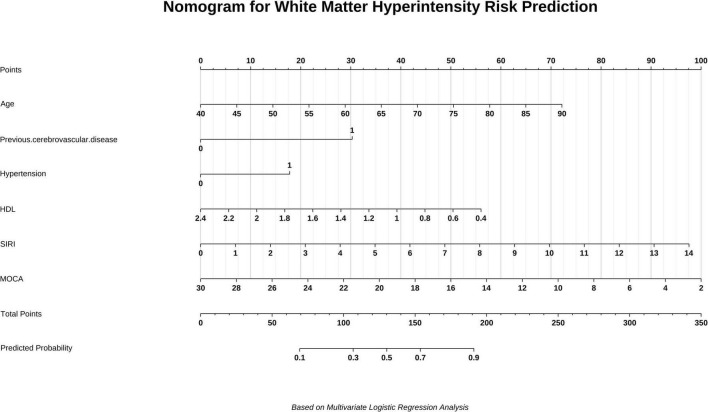
The nomogram used to evaluate WMH burden. The vertical line at the top of the bar chart represents the total score for each independent variable (0–100 points). The total scores of all variables are added to determine the predicted risk value above the bottom of the prediction line. For categorical variables, “0” indicates the absence of disease or condition, and “1” indicates the presence of disease or condition. Among the categorical variables, “0” indicates the absence of previous cerebrovascular disease and hypertension, while 1’ indicates their presence. HDL, high-density lipoprotein; Siri, systemic inflammation marker; MOCA, Montreal Cognitive Assessment Scale.

The association analysis in this study included 14 variables encompassing demographic characteristics, metabolic parameters, and systemic inflammatory markers. To improve model accuracy, age is analyzed as a stratified categorical variable. Variables showing statistical significance (*P* < 0.1) in univariate analysis were entered into a multivariate backward stepwise logistic regression model, with a retention criterion of *P* < 0.05 in the final model. Age, hypertension, HDL-C, SIRI, previous cerebrovascular disease and MoCA score were identified as independent risk factors for moderate to severe WMH occurred (Table 2). Convert the age group variable in the model to numeric. Subsequently, a nomogram was constructed and the predictive model was evaluated (Figure 4).

**TABLE 2:** Univariate and multivariate logistic regression analysis.

Variables	Single factor					Multiple factor				
	β	S.E	Z	P	OR (95%CI)	β	S.E	Z	P	OR (95%CI)
**Age**										
42–58					1.00 (Reference)					1.00 (Reference)
59–75	0.62	0.34	1.83	0.068	1.87 (0.96 ∼ 3.65)	0.24	0.40	0.60	0.552	1.27 (0.58 ∼ 2.76)
76–92	1.48	0.40	3.73	<0.001	4.41 (2.02 ∼ 9.63)	0.98	0.47	2.10	0.036	2.67 (1.07 ∼ 6.70)
Hypertension	0.97	0.23	4.15	<0.001	2.64 (1.67 ∼ 4.17)	0.71	0.28	2.53	0.011	2.03 (1.17 ∼ 3.52)
Previous cerebrovascular disease	1.63	0.29	5.66	<0.001	5.13 (2.91 ∼ 9.04)	1.06	0.33	3.20	0.001	2.89 (1.51 ∼ 5.52)
HDL	–1.27	0.42	–3.03	0.002	0.28 (0.12 ∼ 0.64)	–0.99	0.49	–2.02	0.043	0.37 (0.14 ∼ 0.97)
LDL	–0.33	0.13	–2.53	0.011	0.72 (0.55 ∼ 0.93)					
TC	–0.32	0.11	–2.92	0.004	0.73 (0.59 ∼ 0.90)					
UA	0.01	0.00	2.58	0.010	1.01 (1.01 ∼ 1.01)					
Monocyte	1.87	0.65	2.87	0.004	6.47 (1.81 ∼ 23.11)					
Platelets	–0.01	0.00	–2.35	0.019	0.99 (0.99 ∼ 0.99)					
LWR	–2.55	1.22	–2.09	0.037	0.08 (0.01 ∼ 0.86)					
PNR	–0.01	0.01	–2.44	0.015	0.99 (0.98 ∼ 0.99)					
SIRI	0.36	0.13	2.84	0.005	1.44 (1.12 ∼ 1.85)	0.27	0.14	1.96	0.049	1.31 (1.01 ∼ 1.72)
MOCA	–0.16	0.02	–7.36	<0.001	0.85 (0.81 ∼ 0.89)	–0.13	0.02	–5.62	<0.001	0.87 (0.83 ∼ 0.92)

OR, odds ratio; CI, confidence interval.

### 3.4 Analysis of predictive efficacy of risk factors for moderate to severe WMH patients

**FIGURE 5:**
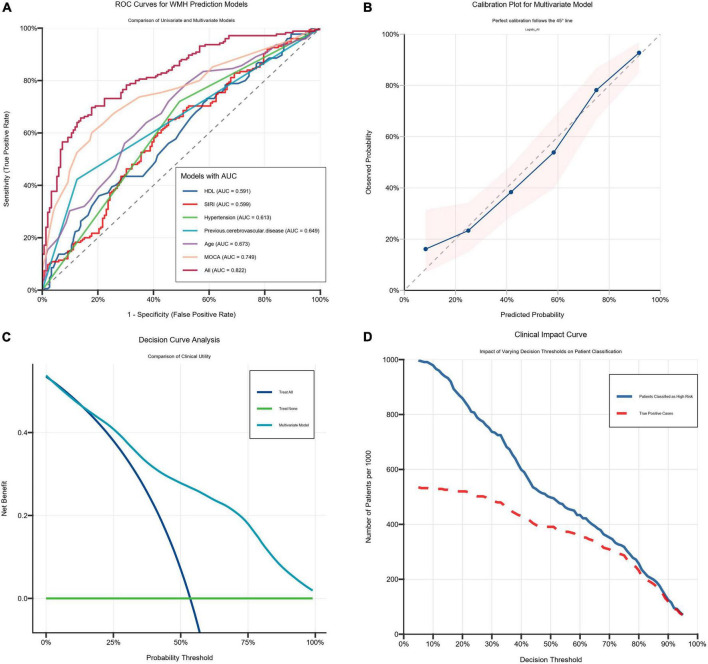
Six variables combined to predict performance. **(A)** The receiver operating characteristic curve (ROCauc) curve six variables combined to predict performance. **(B)** The calibration curve of six variables combined to predict performance. **(C)** The decision curve analysis (DCA) of six variables combined to predict performance. **(D)** The clinical impact curve (CIC) of six variables combined to predict performance.

ROC curve analysis based on the variables from the final model yielded the following results: For SIRI, AUC was 0.599 (95% CI: 0.537–0.660, *P* = 0.002), with a Youden index of 0.197, an optimal cutoff value of 1.016, and corresponding sensitivity and specificity of 65.1 and 54.6%, respectively. For MoCA score, the AUC was 0.749 (95% CI: 0.696–0.802, *P*< 0.001), with a Youden index of 0.422, an optimal cutoff of 19.5 points, and sensitivity and specificity of 60.0 and 82.2%, respectively. The combined predictive model achieved an AUC of 0.822 (95% CI: 0.779–0.868, *P*< 0.001), with a Youden index of 0.528, an optimal cutoff of 0.656, and sensitivity and specificity of 64.6 and 88.2%, respectively. The combined model demonstrated superior predictive performance for moderate-to-severe WMH compared with either marker alone (Figure 5A).

The calibration curve analysis indicated excellent calibration agreement between the model’s predictions and the actual observed outcomes (Figure 5B). DCA showed that within a threshold probability range of 0.05–1.0, the nomogram yielded greater net benefit than both the “treat-none” and “treat-all” strategies (Figure 5C). CIC was used to evaluate the clinical utility of the risk prediction nomogram (Figure 5D). The CIC visually demonstrated superior overall net benefit for the nomogram across a wide range of threshold probabilities and in scenarios impacting patient outcomes, indicating significant predictive value of the model.

### 3.5 Internal validation and model comparison

**FIGURE 6:**
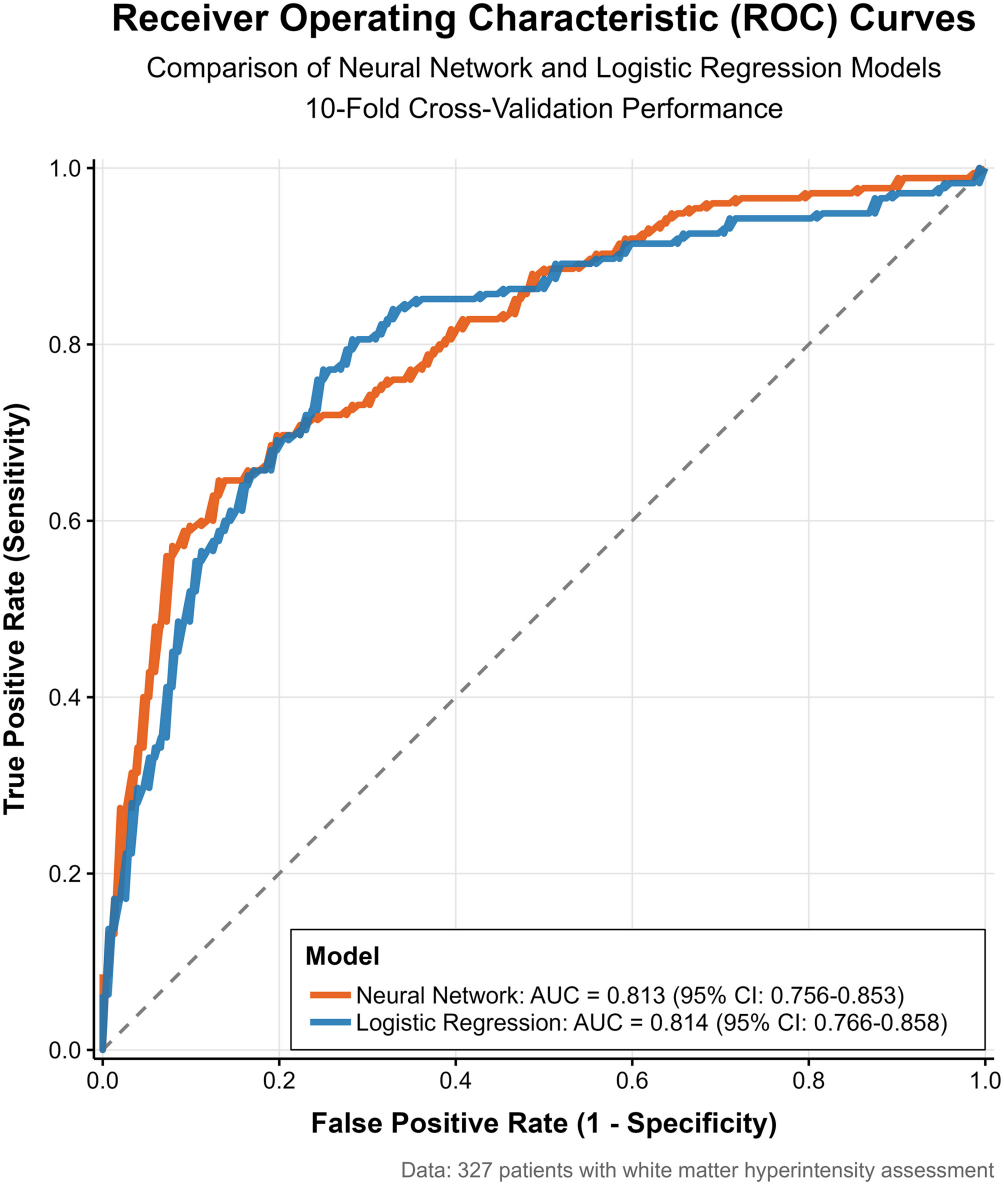
The ROCauc curve of subjects’ working characteristics was used to compare the predictive performance of the two combined models.

Using the independent predictors from the logistic regression model, an artificial neural network was developed (Figure 6). Both models demonstrated comparable discriminatory performance in 10-fold cross-validation (DeLong test, *P* = 0.587), with AUCs of 0.813 (95% CI: 0.756–0.853) for the neural network and 0.814 (95% CI: 0.766–0.858) for the logistic regression model.

Internal validation confirmed that both models maintained good predictive performance and comparable classification accuracy. The neural network further validated the robustness of the logistic regression model. Although the neural network approach offers greater flexibility in capturing complex nonlinear patterns, the logistic regression model provides superior clinical applicability and interpretability while maintaining comparable predictive performance.

### 3.6 Model interpretability

#### 3.6.1 SHAP analysis for logistic regression model

**FIGURE 7:**
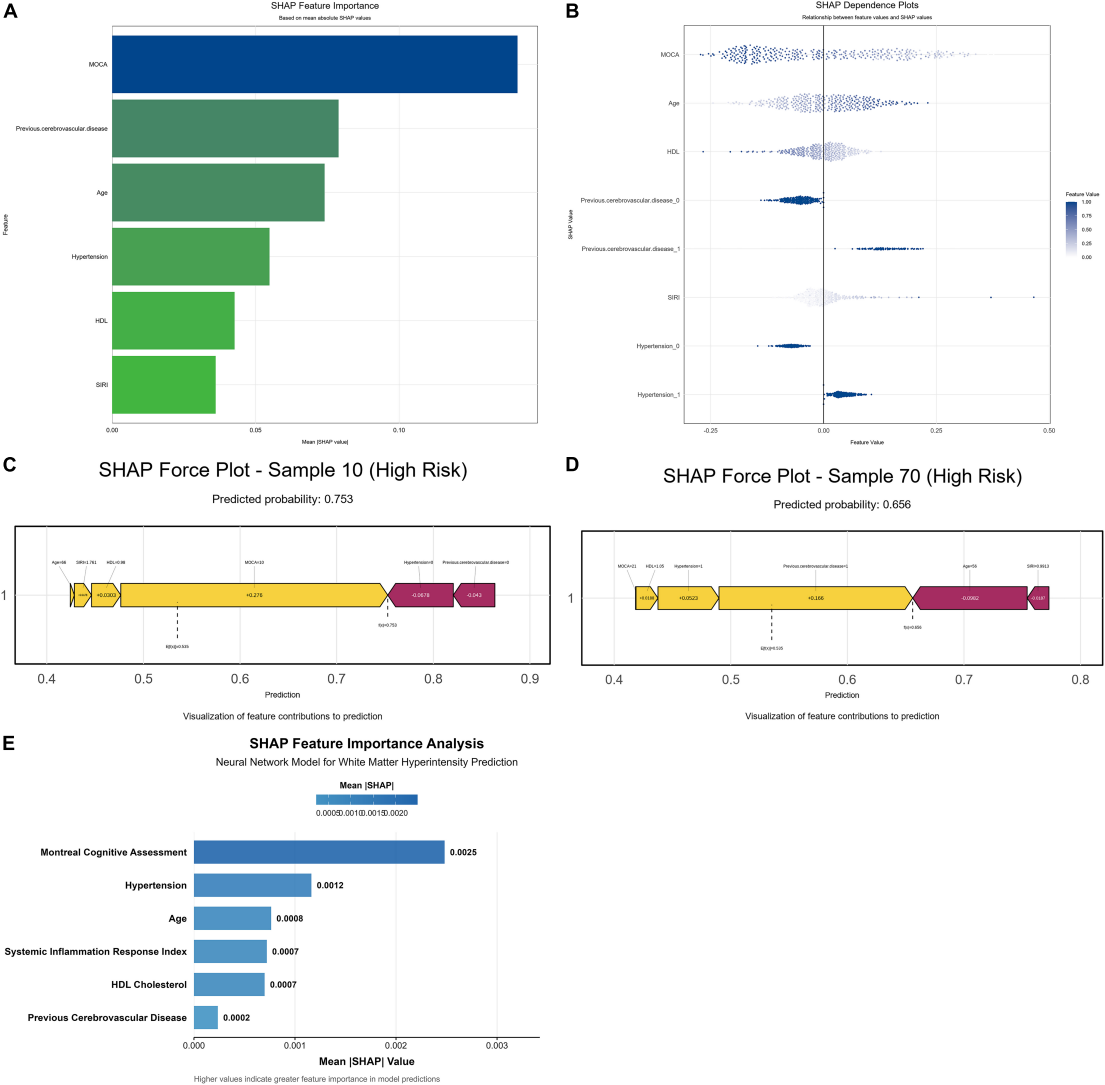
SHAP interpretation of model constructed by human–machine collaboration group. **(A)** Importance chart of SHAP variables, with the included features sorted by the average absolute value of SHAP from highest to lowest. **(B)** Each point represents a feature value, and different colors represent the final influence of the feature on the model output results, where deep color represents a larger value and light color represents a smaller value. **(C,D)** SHAP force plot for two cases: Color indicates the contribution of each feature, red indicates that the feature has a negative effect on the prediction (arrow to the left, SHAP value decreases), and yellow indicates that the feature has a positive effect on the prediction (arrow to the right, SHAP value increases). The length of the color bar indicates the strength of the contribution, and E[*f*(x)] indicates the SHAP reference value, which is the mean predicted by the model. [*f* (x)] represents the SHAP value of the individual. 0 is no and 1 is yes. **(E)** The SHAP variable importance plot of the neural network model, with features sorted by relative SHAP value from highest to lowest.

SHAP analysis identified MoCA score, previous cerebrovascular disease, hypertension, and age as the predominant predictors (Figure 7A). In the SHAP beeswarm plot, data points for hypertensive patients shifted to the right (indicating higher SHAP values), suggesting a stronger contribution to WMH burden (Figure 7B).

Individual SHAP force plots (Figure 7C) illustrated the contribution of each feature: low MoCA score (++0.28 SHAP) and previous cerebrovascular disease (+0.122) increased the predicted risk, whereas a low SIRI score (−0.032) reduced it. These findings confirm the differential impacts of the inflammatory marker (SIRI) and the cognitive factor (MoCA) on WMH risk. Figure 7D further visualizes the contribution of individual features to the model’s decision boundary.

#### 3.6.2 SHAP feature importance analysis for neural network models

To further validate the interpretability of the neural network model, SHAP analysis was performed using the final model trained on the complete dataset. Specifically, 200 instances were randomly sampled to establish the background dataset. SHAP values were then approximated using 50 randomly selected background samples per prediction. The resulting SHAP summary plot identified MoCA, hypertension, age, and SIRI as the primary predictors (Figure 7E).

## 4 Discussion

WMH are common neuroimaging findings in the aging population, whose severity is positively correlated with cognitive impairment, neuropsychiatric symptoms, and increased mortality risk (Chen et al., 2021; Debette et al., 2019; Moroni et al., 2020; Schoemaker et al., 2022). We developed and internally validated two predictive models. Both models demonstrated good discriminatory performance in 10-fold cross-validation (AUC = 0.81). For the current prediction task, the relatively simple logistic regression model adequately captured key predictive relationships, while the more complex neural network did not provide significant performance improvement in internal validation. These findings provide empirical support for selecting more parsimonious and interpretable models in clinical practice. Correlation analyses revealed significant associations between moderate-to-severe WMH and age, hypertension, diabetes, and previous cerebrovascular disease. Among these, hypertension, MoCA score, SIRI, HDL levels, age, and previous cerebrovascular disease were confirmed as independent risk factors for moderate-to-severe WMH. SHAP analysis of both models further identified MoCA, hypertension, and age as key determinants. Our findings highlight several modifiable risk factors for WMH progression. Notably, both blood pressure control and antihypertensive therapy has been consistently shown to slow the progression of WMH (Lai et al., 2020; van Middelaar et al., 2018), underscoring the importance of vascular risk management. At the tissue level, the pathological correlates of WMH, including demyelination and gliosis, represent potential mechanisms through which these vascular risk factors ultimately lead to cognitive impairment. This mechanistic understanding supports the potential value of early cognitive rehabilitation strategies for high-risk WMH patients, though this requires validation in prospective studies. In our cohort, the prevalence of diabetes mellitus was significantly higher in the moderate-to-severe WMH group than in the mild group (*P* < 0.05). However, no significant associations were found between smoking or alcohol and WMH burden, possibly due to the limited sample size. Among participants aged ≥ 40 years, no significant sex-based differences in WMH severity were observed.

### 4.1 Biological significance and predictive value of SIRI in CSVD

Our study demonstrated that SIRI (neutrophil count × monocyte count)/lymphocyte count], derived from neutrophils, monocytes, and lymphocytes, exhibits significant predictive value for moderate-severe WMH and correlates significantly with WMH burden. These findings are consistent with previous research on other inflammatory biomarkers such as NLR (Chuang et al., 2023; Nam et al., 2022; Xiao et al., 2023). Notably, SIRI showed a stronger correlation with PVWMH *(r* = 0.22, *P* < 0.01) than with subcortical WMH (*r* = 0.13, *P* = 0.16), suggesting that inflammatory processes may preferentially affect perivascular regions where the blood–brain barrier is relatively weaker. This finding is consistent with the report by Wang et al. (2021), which linked neutrophil and monocyte counts to outcomes following acute cerebrovascular events.

Prior studies demonstrate that reduced peripheral lymphocyte counts independently predict adverse outcomes post-cerebrovascular events (Gong et al., 2021). We observed a positive correlation between the SIRI and WMH burden (*r* = 0.19, *P* < 0.001). This suggests that SIRI may reflect the balance between adaptive immune responses (indicated by lymphocyte levels) and inflammatory responses (reflected by neutrophil counts), providing new insights into the role of adaptive immunity in WMH pathogenesis. Potential mechanisms may involve lymphocyte-mediated vascular repair through anti-inflammatory cytokines such as IL-10 (Huang et al., 2021), while patients with WMH often exhibit immunosenescence, characterized by lymphopenia and elevated C-reactive protein (CRP) levels (Lian et al., 2020; Soto-Heredero et al., 2023). This immune dysregulation may exacerbate neurovascular injury through SASP, indicating that maintaining immune homeostasis could serve as a novel therapeutic strategy.

The immune system, inflammation, and hypertension are pathophysiologically interconnected (Manzano et al., 2025; Costa et al., 2001). The innate and adaptive immune systems trigger inflammatory processes that contribute to elevated blood pressure and subsequent organ damage (Roy et al., 2022). Cells of the innate immune system produce reactive oxygen species (ROS), including superoxide and hydrogen peroxide, primarily to eliminate pathogens. Prolonged inflammation enhances ROS production, resulting in oxidative stress that induces endothelial dysfunction. The endothelium regulates vascular tone and structure. Persistent inflammation reduces nitric oxide (NO) bioavailability, impairing its vasodilatory function and compromising vascular relaxation. Effector T cells and regulatory lymphocytes—components of the adaptive immune system—contribute to vasoconstriction in hypertension. Signals from the central nervous system and antigen-presenting cells (APCs) activate effector T lymphocyte differentiation, promoting polarization toward Th-1 and Th-17 phenotypes (Ciprandi et al., 2009). Th-1 and Th-17 effector cells drive inflammatory responses that promote WMH development (Ochando et al., 2023).

Compared to indices such as NLR and SII, SIRI provides a more precise reflection of neurovascular inflammation and immune responses by integrating homeostasis information from multiple immune cell lineages. Its principal advantage lies in the comprehensive integration of three key leukocyte subsets, thereby offering a more complete representation of systemic inflammatory status. Unlike single-parameter measurements (e.g., CRP) or two-parameter ratios (e.g., NLR), SIRI simultaneously quantifies neutrophils (representing acute innate immunity), lymphocytes (reflecting adaptive immune regulation), and monocytes (involved in chronic inflammation and immunothrombosis). This integrated approach better captures the complex inflammatory network implicated in WMH pathogenesis, particularly in processes involving endothelial dysfunction and blood-brain barrier disruption.

Our findings align with emerging evidence across various disease domains. For instance, recent studies demonstrate that in statin-treated patients, SIRI shows superior value to traditional biomarkers like high-sensitivity C-reactive protein (hs-CRP) for predicting cardiovascular mortality risk stratification (Xia et al., 2023). Similarly, in metabolic dysfunction-associated steatotic liver disease, SIRI showed better performance in predicting cardiovascular risk than traditional inflammatory indices (Huang et al., 2025). Notably, neither our study nor the regional investigation by Jiang et al. (2022) found significant associations between NLR and WMH, suggesting that geographical or population-specific differences may influence the utility of certain inflammatory markers. Given the high-salt and high-fat dietary patterns in Inner Mongolia, which are more likely to trigger cardiovascular and cerebrovascular diseases compared to other regions, our study included patients with prior cerebrovascular disease. This may be related to early statin prophylaxis in such patients, where the anti-inflammatory and antioxidant effects of statins could normalize NLR levels. Local lifestyle factors and medication practices may also influence model results. Therefore, we recommend SIRI as a complementary biomarker within existing assessment frameworks rather than a replacement for traditional inflammatory markers. Future multi-center cohort studies with external validation could further confirm the model’s generalizability.

### 4.2 Neurobiological mechanisms of cognitive impairment and WMH progression

Our cross-sectional study identified MoCA score as an independent risk factor for Moderate-severe WMH. Multimodal magnetic resonance imaging studies (Wang N. et al., 2023) have demonstrated that WMH disrupt the structure-function coupling, thereby reducing global network efficiency and ultimately leading to cognitive impairment. We used ROC curve to evaluate the ability of patients with different cognitive levels to distinguish WMH patients. Significant differences in cognitive impairment were observed between the mild and moderate-to-severe WMH groups (*P* < 0.001). The MoCA score demonstrated an AUC of 0.749 (*P* < 0.001) for predicting moderate-to-severe WMH, with 60.0% sensitivity and 82.2% specificity. The optimal MoCA cutoff was 19.5, consistent with established thresholds for predicting cognitive impairment and functional decline in clinical practice. This stratification approach assists clinicians in identifying high-risk individuals for targeted interventions. A recent Southeast Asian study (Wang J. D. J. et al., 2023) confirmed the association between white matter hyperintensities and cognitive performance Our study revealed that although PVWMH and DWMH showed similar correlation trends across multiple MoCA subdomains, they exhibited distinct effects on specific cognitive domains. PVWMH demonstrated stronger correlations with attention and delayed recall performance, suggesting its primary effect through impaired information processing speed, which subsequently disrupts task execution and memory retrieval. In contrast, DWMH showed comparable association strengths to PVWMH across naming, language, abstract thinking, and orientation domains without distinct specificity. The relatively weaker and more diffuse pattern of cognitive associations suggests that DWMH’s effects may not be independent but rather involve interactions with other underlying brain pathologies, resulting in complex and distributed cognitive consequences. These findings highlight the potential value of our model in early community-based dementia prevention strategies.

Beyond inflammatory markers, emerging evidence suggests perivascular spaces (PVS) as promising biomarkers for vascular cognitive impairment (Wang et al., 2025). PVS burden correlates with GFAP, Aβ42/40 ratio, and cognitive domains affected in WMH. Future studies should integrate PVS quantification with inflammatory profiles to elucidate glymphatic system involvement in WMH pathogenesis.

### 4.3 The effect of other related factors on WMH burden

The findings indicate that individuals with moderate to severe WMH exhibit lower levels of LDL, HDL, and TC, which may be attributable to multiple factors. First, the moderate-to-severe WMH group likely had a higher prevalence of pre-existing clinical cardiovascular disease, leading to more intensive and long-standing statin therapy. The potent lipid-lowering effects of these widely prescribed medications could significantly confound the observed association. Moreover, our data concurrently showed elevated systemic inflammatory markers (e.g., SIRI, NLR) in the severe group. Chronic low-grade inflammation is known to alter hepatic lipoprotein synthesis and accelerate catabolism, consequently reducing circulating LDL-C and HDL-C levels *(Nyavor and Obeng-Gyasi, 2025). Finally, this is a cross-sectional study, and the observed lipid levels are a single snapshot in time. The lower levels may reflect long-term consequences of the disease process rather than initial risk factors. Higher lipid levels might have been present earlier in the disease course and contributed to WMH pathogenesis, but their levels dropped over time due to the mechanisms above.*

In summary, the lower lipid levels in Moderate-severe WMH cohort are unlikely to be protective but rather may be a consequence of the disease’s severity, associated comorbidities, and treatments. This highlights the complex interplay between lipids, inflammation, and progression in WMH burden.

### 4.4 Exploration of novel biomarkers and clinical transformation pathway

In our study, SIRI demonstrated better predictive performance than traditional markers such as NLR (Chuang et al., 2023). For clinical translation, we propose a stratified management strategy: individuals with a predicted probability ≥65.6% (high risk) should be prioritized for MRI screening, whereas those with a probability < 65.6% (low risk) could be managed with annual follow-up. Our model stratifies patients into low versus high WMH burden groups (Fazekas scores 0–2 vs. 3–6), aligning with established clinical thresholds for predicting cognitive impairment and functional decline. This stratification may help clinicians identify high-risk individuals for targeted interventions. Furthermore, our findings support the exploration of anti-inflammatory strategies aimed at slowing WMH progression and mitigating cognitive decline. Current management strategies primarily focus on controlling risk factors (e.g., hypertension) to slow disease progression, as no disease-modifying WMH therapies exist. These inflammatory mechanisms highlight the therapeutic potential of anti-inflammatory agents like colchicine, currently under investigation as adjunct therapy for secondary prevention in mild ischemic stroke (Kelly et al., 2024; Low et al., 2019). The integration of inflammatory biomarkers into clinical protocols may improve WMH management and contribute to the shift toward precision medicine and individualized therapeutic approaches. Notably, combining anti-inflammatory treatments with complementary strategies may enhance the overall therapeutic efficacy for WMH. Therefore, future research should focus on developing multi-target interventions that simultaneously address hypertension and neuroinflammation (Solé-Guardia et al., 2023), potentially slowing WMH progression and improving patient outcomes.

Longitudinal data from the Fremington Stroke Risk Profile (FSRP) study (Youssef et al., 2024) confirmed that higher FSRP scores (65 years or older, smoking history, systolic blood pressure over 130 mmHg, diabetes, coronary heart disease, atrial fibrillation, left ventricular hypertrophy, and antihypertensive medication use) were associated with accelerated white matter hyperintensity (WMH) progression and cognitive decline. This consistency underscores the potential validity of our identified factors, though our cross-sectional design requires longitudinal confirmation. However, given the limited specificity of most blood-based biomarkers, their clinical utility requires integration with established risk factors and/or more specific laboratory tests to enhance diagnostic accuracy. Second, as this was an observational study with heterogeneous inclusion criteria, potential selection and publication biases should be acknowledged. Finally, cross-sectional studies cannot assess the longitudinal association between biomarkers and WMH. Therefore, multicenter prospective cohort studies are required to validate the model externally, enhance its generalizability, and further establish causal relationships.

## 5 Conclusion

In summary, this study developed and validated a predictive model integrating metabolic parameters with complete blood count-derived inflammatory markers, and systematically demonstrated the combined value of SIRI and MoCA score in predicting moderate-to-severe WMH. Furthermore, we established a clinically-based risk stratification model and conducted mechanistic analysis of the incorporated variables. Future studies should prioritize external validation through multicenter cohort studies, which represents a crucial step for verifying the model’s clinical utility across broader populations. Additionally, exploration of interventions targeting SIRI modulation and in-depth investigation into the mechanistic effects of metabolic and inflammatory factors on WMH progression are warranted.

## Data Availability

The raw data supporting the conclusions of this article will be made available by the authors, without undue reservation.
